# Activity of the Chimeric Lysin ClyR against Common Gram-Positive Oral Microbes and Its Anticaries Efficacy in Rat Models

**DOI:** 10.3390/v10070380

**Published:** 2018-07-20

**Authors:** Jingjing Xu, Hang Yang, Yongli Bi, Wuyou Li, Hongping Wei, Yuhong Li

**Affiliations:** 1 The State Key Laboratory Breeding Base of Basic Science of Stomatology (Hubei-MOST) & Key Laboratory of Oral Biomedicine, Ministry of Education, School of Stomatology, Wuhan University, Wuhan 430079, China; 2015203040023@whu.edu.cn (J.X.); 2014203040013@whu.edu.cn (Y.B.); 2016203040006@whu.edu.cn (W.L.); 2 Key Laboratory of Special Pathogens and Biosafety, Center for Emerging Infectious Diseases, Wuhan Institute of Virology, Chinese Academy of Sciences, Wuhan 430071, China; yangh@wh.iov.cn (H.Y.); hpwei@wh.iov.cn (H.W.)

**Keywords:** dental caries, bacteriophage lysin, biofilm, antibacterial, anticaries

## Abstract

Dental caries is a common disease caused by oral bacteria. *Streptococcus mutans* and *Streptococcus sobrinus* are the primary cariogenic microbes that often survive as biofilms on teeth. In this study, we evaluated the activity of ClyR, a well-known chimeric lysin with extended streptococcal host range, against common Gram-positive oral microbes and its anticaries efficacy in rat models. ClyR demonstrated high lytic activity against *S. mutans* MT8148 and *S. sobrinus* ATCC6715, with minor activity against *Streptococcus sanguinis*, *Streptococcus oralis*, and *Streptococcus salivarius*, which are considered as harmless commensal oral bacteria. Confocal laser scanning microscopy showed that the number of viable cells in 72-h aged *S. mutans* and *S. sobrinus* biofilms are significantly (*p* < 0.05) decreased after treatment with 50 µg/mL ClyR for 5 min. Furthermore, continuous administration of ClyR for 40 days (5 µg/day) significantly (*p* < 0.05) reduced the severity of caries in rat models infected with a single or a mixed bacteria of *S. mutans* and *S. sobrinus*. Therefore, ClyR could be a promising agent or additive for the prevention and treatment of dental caries.

## 1. Introduction

Dental caries remains a significant problem all over the world despite improved oral hygiene awareness [1]. For more than a century, it has been known that caries is initiated by demineralization of the enamel as a result of fermentation and acidogenesis by oral bacteria in biofilm [2]. Among cariogenic microbes, *Streptococcus mutans* is identified as the primary cariogenic pathogen because of its unique acid-producing and aciduric ability [3,4]. Glucan produced by *S. mutans* plays a fundamental role in biofilm formation, providing binding sites for bacteria colonization on enamel surface [5]. *Streptococcus sobrinus*, another important cariogenic microorganism, is less frequently detected than *S. mutans* in the oral cavity but is considered more virulent than *S. mutans* due to its high acidogenicity and acid tolerance [6]. Previous studies have observed that co-existence of both species is associated with higher incidence of dental caries and higher scores in the DMFT (decayed missing and filled teeth) index in children with early childhood caries (ECC) [7,8]. The amounts of oral *S. mutans* and *S. sobrinus* are used as risk indicators for dental caries [9].

There are presently a few ways to reduce dental caries, where fluoride therapy is the most common method to reduce the risk of the disease [10]. However, fluoride toothpaste (with concentrations of 1000 ppm and above) or fluoride supplements may exposure children under six, especially under three to higher risks of dental fluorosis [11], fluoride toxicity [12], and even fatality caused by accidental ingestion (http://fluoridealert.org/articles/kennerly/). Cariogenic biofilms can be removed by traditional nonspecific mechanical brushing or flossing or by rinsing with a broad-spectrum antibiotic such as chlorhexidine. Derivatives and extractives from natural products, such as fractions of barley coffee [13], cranberry constituents [14], and α-mangostin with/or lawsone methyl ether [15], have been shown to prevent *S. mutans* biofilm formation. In addition, numerous small molecules, including chitosan, 2-amino-imidazole/triazole conjugate [16], and apigenin [17], have shown good antibiofilm activity. However, few of these treatments confer selectivity against *S. mutans* biofilms. In recent years, antimicrobials capable of selectively eliminating *S. mutans* have been designed to achieve targeted killing with minimal effect on other oral microbes [18,19]. This has opened up alternative methods to prevent caries without disturbing the ecological balance to commensal bacteria in the oral cavity [20].

Endolysins are bacteriophage-encoded enzymes that degrade bacterial peptidoglycan at the end of the lytic cycle. Exogenous application of recombinant endolysins to Gram-positive bacteria rapidly induces osmotic lysis and, consequently, cell death. In recent years, endolysins and their derivatives have emerged as a novel class of antibacterial [21,22], and some of them have now entered clinical trial phases [23,24]. Many lysins are reported to be superior to antibiotics in biofilm eradication [25,26]. Their rapid mode of action and specificity differentiate them from antibiotics. Another advantage of endolysins is that they have a low risk of resistance because they target the essential conserved elements on bacterial cell walls that maintain cell viability [27,28].

Recently, our cooperator engineered a chimeolysin ClyR (with its catalytic domain from the CHAP domain of PlyC lysin and the cell wall binding domain from PlySs2 lysin), which was found by an induced lysis-based rapid screening method with broad activity against various Gram-positive pathogens, including multiple strains of streptococci, part of staphylococci, and enterococci [29]. The bactericidal activity of ClyR against *S. mutans* has been further demonstrated in vitro and in a dental colonization mouse model previously [30], however, its bactericidal activity and anticaries efficacy against mixed oral microbes still need to be established. In this study, we determined the efficacy of ClyR against multiple oral Gram-positive bacteria, including *S. sobrinus* and *S. mutans* isolated from children with severe early childhood caries (SECC) in China. The anticaries efficacy of ClyR was further investigated in rat models infected with a single or mixed bacteria of *S. mutans* and *S. sobrinus*.

## 2. Materials and Methods

### 2.1. Bacterial Strains

*S. mutans* MT8148, *S. sobrinus* ATCC6715, *Streptococcus oralis* ATCC10557, *Streptococcus salivarius* ATCC7073, and *Streptococcus sanguinis* ATCC10556 were supplied by the Key Laboratory of Oral Biomedicine Ministry of Education, School of Stomatology, Wuhan University. The clinical strains of *S. mutans* and *S. sobrinus* were isolated from dental plaques of different children with SECC, which were confirmed by biochemistry identification (Tianhe Microbial, Inc., Hangzhou, China) and 16S rDNA sequencing analysis (the primers used were 27F: 5′-AGAGTTTGATCATGGCTCAG-3′; 1492R: 5′-TAGGGTTACCTTGTTACGACTT-3′). The isolates were also genotyped by arbitrarily primed polymerase chain reaction (AP-PCR) [31]. The animal experiment was approved by the Medical Ethics Committee of School of Stomatology of Wuhan University (approval no. 2016-30, 26 February 2016). All streptococci were routinely cultivated in brain heart infusion (BHI) broth (Alpha Bioscience) and grown at 37 °C. For production of biofilms, BHI medium was supplemented with 1% sucrose (BHIS). Luria broth (LB) was used to cultivate *Escherichia coli*.

### 2.2. Cloning, Expression, and Purification of ClyR

The *Escherichia coli* BL21(DE3) strain containing plasmid pET28a-*clyR*, originated from Wuhan Scithera Microbial Technologies Co Ltd. (Wuhan, China), expresses a chimeolysin consisting of the CHAP catalytic domain of PlyC (PlyCAC, amino acids 314–465) and the cell wall binding domain of PlySs2 (PlySb, C-terminal 99 amino acids). As detailed by Yang et al. [29], *E. coli* strain was cultured to an optical density (OD_600_) of ~0.6, induced by adding IPTG to 0.2 mM, and allowed to grow overnight at 16 °C. Purification was performed following the instructions of HisTrap FF columns (GE Healthcare, Chicago, IL, USA). Briefly, columns loaded with sample were washed and eluted with 20 and 250 mM imidazole, respectively. After being dialyzed against phosphate-buffered saline (PBS, containing 137 mM NaCl, 2.7 mM KCl, 4.3 mM Na_2_HPO_4_, 1.4 mM KH_2_PO_4_, pH 7.4), the purified protein was passed through a Detoxi-Gel™ Endotoxin Removing Gel (Thermo Scientific, Waltham, MA, USA) and quantified by a ToxinSensor Chromogenic LAL Endotoxin Assay Kit (GenScript, Nanjing, China) to confirm a low level of endotoxin (<0.2 EU/mL). After quantitation by Bradford assay, the protein solution was stored at −20 °C until use.

### 2.3. Bactericidal Assay

In order to know the lytic activity of ClyR against oral common streptococci, a screening experiment was performed. *S. mutans* MT8148, *S. sobrinus* ATCC6715, *S. oralis* ATCC10557, *S. salivarius* ATCC7073, and *S. sanguinis* ATCC10556 were grown to log-phase at 37 °C and adjusted to an OD_600_ of ~0.8 with PBS buffer as measured in 96-well plates (NEST, Nanjing, China) using a Synergy H1 microplate reader (BioTek, Winooski, VT, USA). After mixing 190 μL of bacterial suspension with 10 μL of the lysin (1 mg/mL) or PBS in a 96-well plate, the drop of OD_600_ in each well was monitored immediately by the microplate reader for 60 min at 37 °C. Meanwhile, viable cell numbers after each treatment were determined by serial ten-fold dilution and plating on BHI agar.

To determine the dose-dependent and time-dependent lytic activity of ClyR, *S. mutans* MT8148 and *S. sobrinus* ATCC6715 resuspended in PBS were adjusted to an OD_600_ of ~0.8. Aliquots of 190 µL of the cell suspension was distributed in 96-well microplates and treated with 10µL of ClyR at different concentrations (0, 25, 50, and 100 µg/mL) for 10 min or 50 µg/mL ClyR for 0, 1, 2, 3, and 5 min at 37 °C. At the end of the reaction, 10-fold serial dilutions of each well were plated on BHI agar and incubated at 37 °C. Resultant colonies were enumerated after 18 h. The bactericidal effect was expressed as the difference in colony-forming units (CFU) from PBS-treated and ClyR-treated groups. To rule out degradation or aggregation of ClyR by salivary proteins, we also tested the activity of ClyR in human saliva. *S. mutans* MT8148 resuspended in PBS buffer or human saliva were treated with 25 µg/mL of ClyR for 1 h at 37 °C, then serially diluted and plated onto BHI agar for CFU enumeration. All experiments were performed independently three times in triplicate.

### 2.4. ClyR Activity against Clinical S. mutans and S. sobrinus Isolates

Clinical *S. mutans* and *S. sobrinus* isolates were grown overnight and brought to an OD_600_ of ~0.8 with PBS. From these bacterial stocks, 190 μL were added to each well of a 96-well microplate. For each strain, each of the well received 10 μL of ClyR at 1 mg/mL (10 μg, resulting in a final concentration of 50 μg/mL). Corresponding triplicate wells received 10 μL of PBS as controls. The Synergy H1 microplate reader was used to measure the drop of OD_600_ of the mixture at 37 °C for 10 min.

### 2.5. Transmission Electron Microscopy (TEM)

TEM was used to visualize the effects of ClyR on the bacterial cell wall. Log phase *S. mutans* MT8148 and *S. sobrinus* ATCC6715 were washed with PBS and adjusted to an OD_600_ of 0.8, then treated with 50 µg/mL ClyR or PBS at 37 °C for 5 min and resuspended in PBS. After fixation by 2.5% glutaraldehyde, samples were analyzed by a transmission electron microscope (Tecnai G^2^ 20 Twin; Fei, Hillsboro, OR, USA).

### 2.6. Scanning Electron Microscopy (SEM)

*S. mutans* MT8148 and *S. sobrinus* ATCC6715 were inoculated into BHIS in a 6-well plate with a glass coverslip in each well. After incubation for 72 h, biofilms formed on the glass coverslips were rinsed three times with sterile PBS to remove planktonic and loosely adherent cells. The biofilms were treated with either 1 mL of 50 μg/mL ClyR or PBS for 5 min simultaneously, followed by prefixing with 2.5% glutaraldehyde for 10 min before fixation in 1% osmium tetroxide for 1 h. After that, the coverslips were subjected to a series of ethanol (30%, 50%, 60%, 70%, 80%, 90% and 100%) for dehydration. Finally, samples were coated with gold (Hummer VI; Technic Inc., Anaheim, CA, USA) before observation under SEM (SEM, SU8010, Hitachi, Japan) at an accelerating voltage of 5 kv using 5000× and 10,000× magnifications.

### 2.7. Confocal Laser Scanning Microscopy (CLSM)

The 72-h biofilms exposed to 50 μg/mL of ClyR or PBS for 5 min were also stained by LIVE/DEAD^®^ Bac-Light^TM^ Bacterial Viability kit L-7012 (Invitrogen, Carlsbad, CA, USA) for 15 min and washed twice with PBS. The stained biofilms were viewed using a confocal laser scanning microscope (UltraVIEW VoX; PerkinElmer, Waltham, MA, USA) at a 1024 × 1024 pixel scan area using a 60× lens. Four random areas of each biofilm on each coverslip were scanned. A stack of 10–20 slices in 0.5 μm step sizes was captured from the top to the bottom of the biofilm. All acquired images were analyzed by the Volocity (version 6.3.0, PerkinElmer, Waltham, MA, USA) software supplied with the instrument.

### 2.8. Quantify Recovered Biofilm Bacteria in Vitro

To determine the viable cell number within biofilm after ClyR treatment, 10 µL overnight culture of *S. mutans* MT8148 or *S. sobrinus* ATCC6715 was mixed with 250 µL BHIS in a 48-well polystyrene plate (Tissue culture treated, Nest, China), and incubated at 37 °C for 72 h to allow biofilm formation. After removing the supernatant and washing twice with sterile PBS, biofilms were treated with 200 µL of 50 μg/mL ClyR or PBS for 5 min. After washing twice with PBS, adherent cells from the biofilm were resuspended by vigorous pipetting and vortexing and, finally, serially diluted and plated on BHI agar for CFU enumeration.

### 2.9. Animal Study

Rat models were used to evaluate the anticaries activity of ClyR in vivo as previously described with some modifications [20]. Thirty six female Sprague Dawley (SD) rats (21 days old) were purchased from Hubei Medical Laboratory Animal Center (Wuhan, China). Animals were housed in the State Key Laboratory Breeding Base of Basic Science of Stomatology (Hubei-MOST) and Key Laboratory of Oral Biomedicine of Ministry of Education (KLOBM) under specific pathogen-free conditions. All animal experiments were conducted with the approval of the Animal Experiments Committee of Wuhan University (approval no. 2016-72). The rats were fed with Keyes 2000 cariogenic diet supplemented with antibiotics (1.0 g/kg each of ampicillin, chloramphenicol, and carbenicillin) and water containing 4000 U/mL penicillin G for the first 3 days to temporarily suppress the proliferation of oral flora to facilitate bacterial infection. At day 25, 36 SD female rats were randomly divided into three groups and infected with 200 µL overnight cultures of *S. mutans* MT8148, *S. sobrinus* ATCC6715, or a mixture of *S. mutans* MT8148 and *S. sobrinus*, respectively, for the next 6 days. At day 31, half of the rats in each group were treated by pipetting 100 µL ClyR (50 μg/mL) solution per day into the mouth of each rat for 40 days continuously; the rest of the rats were treated with PBS as control. All the rats were euthanized at day 120. After removal of flesh from the jaws, the teeth were observed by stereomicroscope (Olympus, Tokyo, Japan) and stained for caries scoring by Keyes’ method [32].

### 2.10. Statistical Analysis

Experimental data are presented as means ± standard errors of means. One-way ANOVA test is performed for the statistical analysis. *p* value of <0.05 is considered to be statistically significant.

## 3. Results

### 3.1. High Bactericidal Activity of ClyR against Planktonic S. mutans and S. sobrinus

**Figure 1:**
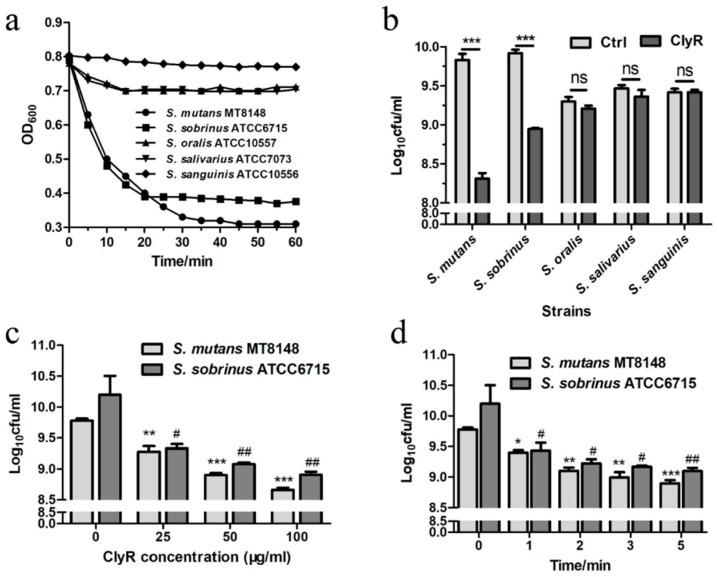
Activity of ClyR against oral microbes. (**a**) Activity of ClyR against common oral bacteria. *S. mutans* MT8148, *S. sobrinus* ATCC6715, *S. oralis* ATCC10557, *S. salivarius* ATCC7073, and *S. sanguinis* ATCC10556 were treated with 50 µg/mL ClyR for 1 h; the Y-axis represents the bacteria turbidity at optical density (OD_600_); (**b**) Viable cell number after each treatment was determined by serially ten-fold dilution and plating on brain heart infusion (BHI) agar; (**c**) Dose-dependent bactericidal activity of ClyR. *S. mutans* and *S. sobrinus* were treated with different concentrations of ClyR (0, 25, 50, and 100 µg/mL) for 10 min, viable cell numbers were determined by plating to BHI agar; (**d**) Time-dependent bactericidal activity of ClyR. Bacteria were treated with 50 µg/mL of ClyR for 0, 1, 2, 3, and 5 min, viable cell numbers were determined by plating on BHI agar. ns: not significant; ^#^ and *: *p* < 0.05; ^##^ and **: *p* < 0.01; ***: *p* < 0.001 compared to the control group.

The screening experiment showed that the major cariogenic bacteria *S. mutans* MT8148 and *S. sobrinus* ATCC6715 are susceptible to ClyR, with rapid OD_600_ decrease from 0.8 to 0.3 or 0.4, after treatment with 50 µg/mL ClyR for 1 h (Figure 1a). A corresponding ~1.5 logs or ~1 logs decrease in cell viability was achieved (Figure 1b). However, minor lytic activity against *S. oralis* ATCC10557, *S. salivarius* ATCC7073, and *S. sanguinis* ATCC10556 was observed (Figure 1a,b). These bacteria are believed to be harmless, normal oral bacteria. Then, the dose-dependent (Figure 1c) and time-dependent (Figure 1d) killing efficacy of ClyR were performed. As shown in Figure 1c, ClyR was highly active against *S. mutans* and *S. sobrinus*, with reduction of 92.37 ± 1.19% and 93.33 ± 3.41% bacteria, respectively, after treatment with 100 µg/mL of ClyR for 10 min. Notably, a rapid mode of action was observed in the first 5 min, with only 13.18 ± 1.1% *S. mutans* and 7.94 ± 2.9% *S. sobrinus* remaining after treatment with 50 µg/mL of ClyR (Figure 1d). Moreover, the activity of ClyR was rare affected by human saliva (Figure S1). Because lysin is intended to be applied directly in mouth, it is preferred that ClyR could kill utmost bacteria in a short time under a low concentration. Therefore, a relatively low ClyR concentration (50 µg/mL) and short treatment time (5 min) were chosen for the biofilm experiments shown below.

### 3.2. Broad Lytic Activity

**Figure 2:**
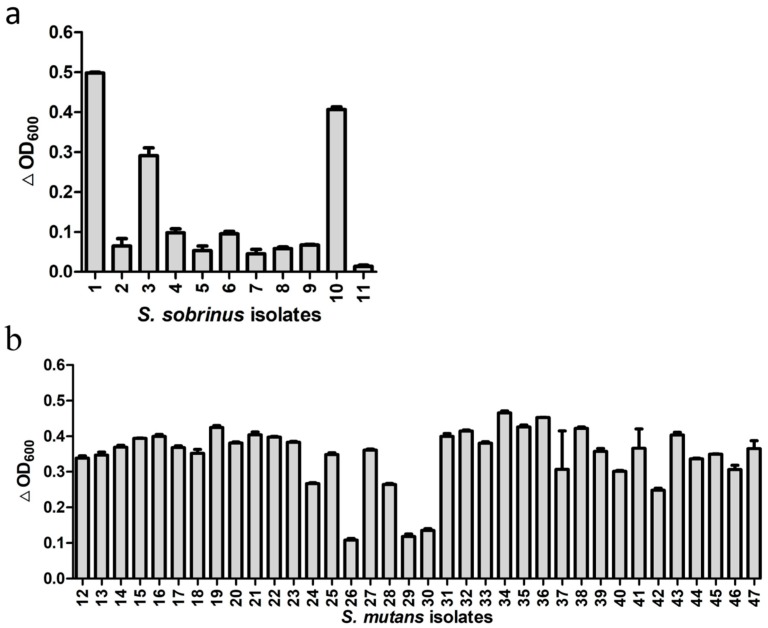
Activities of ClyR against multiple clinical isolates. *S. sobrinus* (**a**) and *S. mutans* (**b**) isolates were grown to logarithmic phase, suspended in PBS buffer to an OD_600_ of ~0.8, and then treated with 50 µg/mL of ClyR at 37 °C for 10 min. The relative change in OD_600_ (∆OD_600_) of each well was determined by subtracting the decrease of OD_600_ in the PBS-treated control well.

Besides the standard strains, we also tested activity of ClyR against 11 *S. sobrinus* isolates from dental plaque samples of eight children with SECC and 36 *S. mutans* isolates from 22 children with SECC. As shown in Table S1 and Figure S2, 8 genotypes of *S. sobrinus* and 27 genotypes of *S. mutans* were identified in these isolates, suggesting the diversity of these isolates. The lytic assay (Figure 2) showed that ClyR has varied activities against these isolates, with 3 of the 11 *S. sobrinus* (Figure 2a) and 33 of the 36 *S. mutans* (Figure 2b) isolates found to be highly susceptible, causing significant drop in OD_600_. The minimum bactericidal concentration (MBC) values (Table S2) of *S. mutans* and *S. sobrinus* isolates ranged from 125 to >1000 μg/mL. Among them, 42 isolates were completely killed with 500 μg/mL or even lower ClyR concentration, while the MBCs of *S. mutans* MT8148 and *S. sobrinus* ATCC6715 were 1000 μg/mL.

### 3.3. TEM Analysis of S. mutans and S. sobrinus Exposed to ClyR

**Figure 3:**
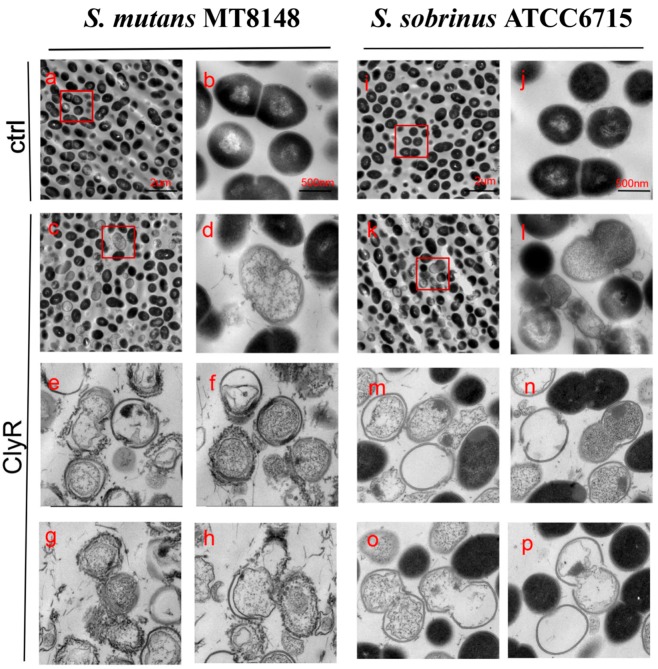
TEM images of *S. mutans* and *S. sobrinus* exposed to ClyR. Bacterial cells were treated with PBS (Ctrl, **a**,**b**,**i**,**j**) or 50 µg/mL of ClyR (**c**–**h**,**k**–**p**) at 37 °C for 5 min and then fixed by 2.5% glutaraldehyde.

TEM Images showed that intact *S. mutans* MT8148 and *S. sobrinus* ATCC6715 cells have normal cellular morphology with integral peptidoglycan layers (Figure 3a,b,i,j). However, when cells were exposed to ClyR, distinct localized degradation of the cell wall was observed, resulting in the loss of their cytoplasmic contents (Figure 3c–h,k–p).

### 3.4. Activity of ClyR against S. mutans and S. sobrinus Biofilms

**Figure 4:**
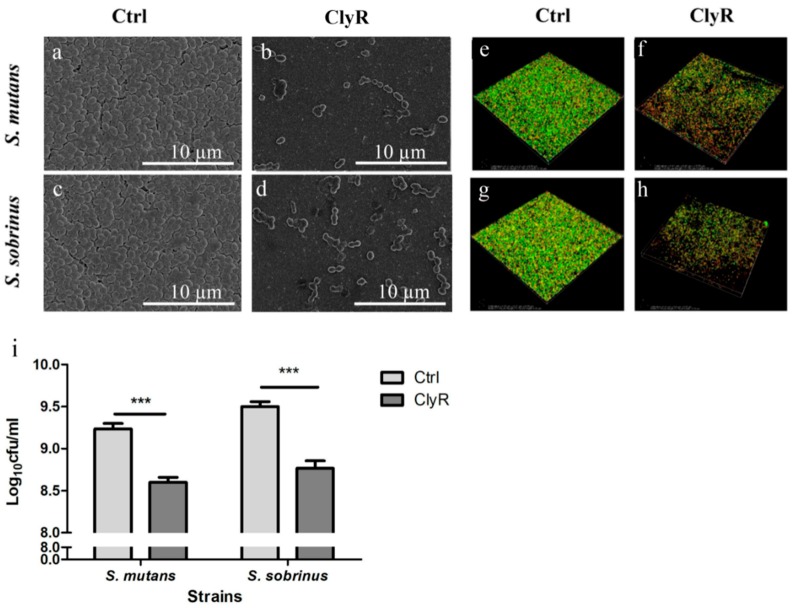
Effect of ClyR on *S. mutans* and *S. sobrinus* biofilms. (**a**–**d**) SEM analysis of bacterial biofilms after treatment with ClyR. 72-h biofilms of *S. mutans* MT8148 and *S. sobrinus* ATCC6715 were treated with PBS (Ctrl) or 50 µg/mL of ClyR for 5 min and then analyzed by SEM; (**e**–**h**) CLSM analysis of bacterial biofilms after treatment with ClyR. 72-h biofilms were analyzed by CLSM to calculate the viable cell percentage after treatment with PBS (Ctrl) or 50 µg/mL of ClyR for 5 min; (**i**) Effect of ClyR on viable cell number within biofilms. 72 h *S. mutans* MT8148 and *S. sobrinus* ATCC6715 biofilms were treated with 50 µg/mL ClyR for 5 min, viable cell number after each treatment was determined and compared with that of the PBS-treated controls (Ctrl). Data is shown as mean ± standard deviation, and *** *p* < 0.001.

The impacts of ClyR on *S. mutans* and *S. sobrinus* biofilms were investigated by SEM, CLSM, and direct CFUs counting assay. The SEM images in Figure 4a–d showed that viable bacteria in 72-h biofilms decreased significantly after treatment with 50 µg/mL of ClyR for 5 min compared with those of the PBS-treated control biofilms. Morphologically, cells after ClyR treatment exhibited an irregular shape and cell wall damage, indicating that ClyR may remove biofilms through its bactericidal activity.

To test whether ClyR affects the viability of *S. mutans* and *S. sobrinus* cells within the biofilm, we performed the CLSM analysis by staining live bacteria with green fluorescence and dead bacteria with red fluorescence. As shown in Figure 4e–h, after treatment with PBS (Figure 4e,g), the percentages of viable bacterial cells within 72 h *S. mutans* and *S. sobrinus* biofilms were 88.10 ± 1.23% and 87.66 ± 1.27%, respectively, and the average thicknesses of the corresponding biofilms were 11.5 ± 2.50 µm and 9.75 ± 0.88 µm, respectively. Meanwhile, after treatment with 50 µg/mL ClyR for 5 min (Figure 4f,h), the percentages of viable bacterial cells were decreased to 47.49 ± 10.34% and 47.01 ± 10.77% for *S. mutans* and *S. sobrinus* biofilms, respectively, with the average thicknesses of the biofilms of 8.40 ± 1.52 µm and 7.75 ± 1.75 µm, respectively. All these reductions were significant (*p* < 0.05) compared with the PBS-treated control groups (Figure 4e,g).

In accordance with the CLSM results, direct CFUs counting assay (Figure 4i) showed that ClyR decrease the number of viable cells in the *S. mutans* and *S. sobrinus* biofilms to 32.51 ± 1.33% and 25.41 ± 1.52%, respectively, compared with the PBS controls (*p* < 0.001).

### 3.5. Anticaries Efficacy of ClyR in Rat Models

**Figure 5:**
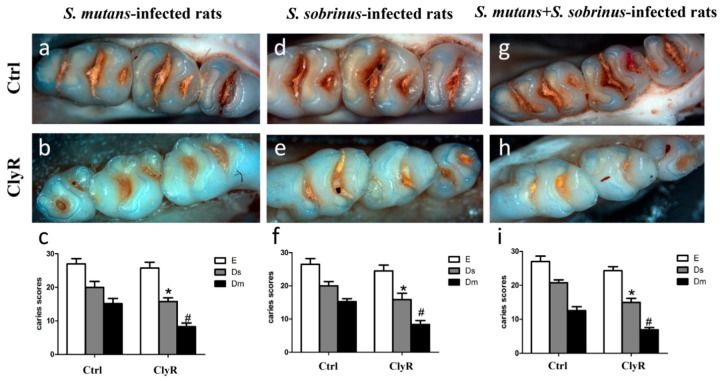
Effect of ClyR on the development of dental caries in rats (representative teeth images and Keyes’ score). Rats were infected with *S. mutans* (**a**–**c**), *S. sobrinus* (**d**–**f**) or a mixture of *S. mutans* and *S. sobrinus* (**g**–**i**) for six days, then treated with 50 μg/mL of ClyR or PBS (Ctrl) for 40 days. The teeth images were recorded and Keyes’ score were calculated on day 120 (**a**,**b**,**d**,**e**,**g**,**h**); Magnification: ×50; E, enamel caries; Ds: dentin exposed; Dm: 3/4 of the dentin affected. * and ^#^: *p* < 0.05 compared to control group.

Our earlier study [30] showed that repeated use of ClyR for 21 days (5 µg/day) significantly reduce the number of colonized *S. mutans* cells in the dental plaques and has no harmful effects on the mice. In the present study, we applied 5 µg/day of ClyR in a longer time (40 days) to further demonstrate its anticaries efficacy in rat models. As shown in Figure 5, severe carious lesions were observed in the PBS-treated control groups (Figure 5a,d,g), while, in ClyR-treated groups, mild occlusal caries were occurred in both single-bacterial and mixed-bacterial infected rat models (Figure 5b,e,h). As for the caries scores, ClyR treated rats infected with *S. mutans* (Figure 5c), *S. sobrinus* (Figure 5f), or a mixture of *S. mutans* and *S. sobrinus* (Figure 5i) demonstrated 25%, 25%, or 33.33% of the dentinal slight (Ds) and 55.56%, 46.67%, or 50% dentinal moderate (Dm) lesions reduction, compared with the PBS-treated groups (*p* < 0.05). However, no significant difference was observed in enamel (E) lesions in all three groups.

## 4. Discussion

In the last two decades, the bactericidal efficacy of endolysins and their derivatives have been proven in different animal models [33,34] and foods [35]. These insights have paved the road for the clinical development of endolysins, with a few leads already in different phases of preclinical and clinical trials. In a recent comparative analysis, Czaplewski and colleagues ranked endolysins as the alternative class of antibacterial with the greatest potential based on their clinical impact and technical feasibility (reviewed in Reference [36]). Therefore, the development of endolysins as antibacterials accommodates an important societal need as antibiotic-resistant bacteria have been emerging and spreading, while the antibiotic development pipeline is seeing a significant decline. A unique feature of endolysins is their specificity. The results of our study showed that ClyR has high antibacterial activity against the two major cariogenic pathogens *S. mutans* and *S. sobrinus* but low activity against the harmless commensal *S. sanguinis*, *S. oralis*, and *S. salivarius* (Figure 1a,b). In our subsequent study, we have assessed ClyR’s lytic activity against several other important oral non-streptococci, including *Enterococcus faecalis* ATCC51299. Its activity against *E. faecalis* (the main pathogen of refractory periapical endodontic lesions) was robust [37]. The normal flora usually play an important role in maintaining oral health. For example, even in a relatively low pH environment, *S. sanguinis* and *S. salivarius* can produce ammonia and other alkaline substances using their own urease or arginine deiminase. This reduces the rapid decline in the pH of the plaque, thereby lowering the incidence of caries [38,39]. Meanwhile, *S. sanguinis* can antagonize certain periodontopathogens by delaying its colonization, which is beneficial to prevent periodontal disease [40]. Moreover, noncariogenic flora have been shown to inhibit, and even prevent, exogenous *S. mutans* colonization in vitro and in vivo [41]. In contrast to current broad-spectrum antibiotics, this specificity minimizes the damage to the ecological balance between commensal residents and pathogens in the oral cavity. In addition to standard strains, we also tested the susceptibility of multiple clinical isolates of *S. mutans* and *S. sobrinus* to ClyR (Figure 2). ClyR showed good activities against most *S. mutans* isolates and 3 out of 11 *S. sobrinus* isolates (1, 3, 10), but minor to no activity against the other 8 *S. sobrinus* isolates. The detailed mechanism behind the tolerance is currently unknown, one possible reason may be that the differences in structure or composition of the bacterial cell wall either prevents lysin from getting access to the peptidoglycan or attenuates the affinity of lysin for its binding or catalytic substrates [42]. The varied susceptibility of *S. mutans* and *S. sobrinus* isolates to ClyR and the relevant mechanism still needs further study.

Cariogenic biofilm is mainly composed of oral bacteria including cariogenic bacterium *S. mutans* and *S. sobrinus*, exopolysaccharide, and bacterial debris [43]. Generally, biofilms can tolerate to antimicrobial agents in two ways: the physical barrier and the bacterial communication. The former is formed by the extracellular matrix, which hinders the penetration of antimicrobial agents into the biofilm [44]; the latter involves stimulating bacteria to produce enzymes and proteins to maintain homeostasis in the microbial community [45]. The bactericidal concentration of antibiotics for biofilm may be 100–1000 folds higher than that for planktonic bacteria. However, our study showed that there is only a minor difference in bactericidal concentrations for ClyR to remove biofilm and planktonic *S. mutans* and *S. sobrinus* cells, which indicates that ClyR might be able to penetrate the biofilms. Since oral biofilms are much more complicated than the models studied here, further study is needed to assess the efficacy of ClyR in real oral environments.

Considering the fact that co-existence of *S. mutans* and *S. sobrinus* is an important risk factor in the development of dental caries [46] and that mixed bacteria colonization is more likely to mimic the physiological condition of oral cavity, we evaluated the protective efficiency of ClyR in rats infected with *S. mutans* and *S. sobrinus* separately or together. To our surprise, the protective efficacies in these three groups were almost the same. ClyR-treated rats demonstrated fewer Ds and Dm lesions, indicating that ClyR possesses the ability to lower the severity of dental caries (Figure 5). We speculated that *S. mutans* and *S. sobrinus* are partly killed by ClyR and the formations of oral biofilms containing these two bacteria are also reduced after the treatment, which in turn lowered the formation of carious lesions. Compared with other treatments, a reduction in total caries (total score = score of E + Ds + Dm) of 24% was observed after treatment with ClyR in *S. mutans* infected rats model (Figure 5c), which is among the best efficacies achieved so far. For example, recently reported glucosyltransferase (Gtf) inhibitor [47] and proanthocyanidins (PAC) [14] showed about 26% and 17% reduction in total caries, respectively. In addition, we did not observe any adverse effects in our animals. Based on its robust lytic activity (Figure 1d) and good safety profile, ClyR might be a promising additive in mouth rinse, toothpaste, or oral spray to prevent caries. However, how to maintain the high activity of ClyR in different formulations still needs to be considered in the future.

In conclusion, we report here that the chimeric lysin ClyR is capable of killing *S. mutans* and *S. sobrinus* under both planktonic and biofilm conditions, with rare effect on other harmless commensal oral bacteria. Good anticaries efficacy of ClyR is further demonstrated in *S. mutans*- and/or *S. sobrinus*-infected rat models. Taken together, ClyR could be a promising agent or ingredient for dental caries prevention and long-term dental treatment. Its potential remains to be further explored.

## Supplementary Materials

Supplementary materials can be found at http://www.mdpi.com/1999-4915/10/7/380/s1.
