# Supplementary material for: Activity of the Chimeric Lysin ClyR against Common Gram-Positive Oral Microbes and Its Anticaries Efficacy in Rat Models
Source: Viruses. 2018 Jul 20;10(7):380. doi: 10.3390/v10070380 (PMC6070986; doi:10.3390/v10070380)

Supplementary Materials

Activity of phage lysin ClyR against common Gram-positive oral microbes and its anti-caries efficacy in rat models

Supplementary materials and methods

Bacteria DNA extraction. 5 ml overnight cultures of *S. mutans* and *S. sobrinus* isolates were harvested and washed with 1ml of TE buffer (10 mM Tris base, 1 mM EDTA, pH 8.0). Then the cells were resuspended in 100 μl of TE, 50 μl of 10% sodium dodecyl sulfate was added and cells were incubated at 65°C for 30 min. The suspension was centrifuged (2000g, 5 min) and the supernatant was removed. After that, the tubes containing the cells were placed in a microwave oven (490 W) and heated for 3 min. The pellets were dissolved in 250 μl of TE and the tubes were frozen at -20°C. Before arbitrarily primed polymerase chain reaction (AP-PCR) assay, the suspension was melted, centrifuged and the supernatant was used in AP-PCR.

AP-PCR assay. AP-PCR was performed using primer 5'-AGGGGTCTTG-3' (OPA5). PCRs were carried out in a 25 μl reaction mixture containing 30-40 ng DNA template, 0.2 mM dNTPs, 0.2 μM primer, 1×*Easy Taq*® Buffer and 2.5 U *Easy Taq*® DNA polymerase (Transgen biotech, China) according to the manufacturer' recommendations. DNA amplification was performed in a thermalcycler (T100, Bio-Rad, USA) with an initial denaturation at 94°C for 5 min, followed by 35 cycles

of denaturation (94℃, 1 min), annealing (36℃, 2 min) and extension (72℃, 2 min). The final extension step was at 72℃ for 7 min. PCR products were analyzed by electrophoresis with 1.5% agarose gel containing 1/10000 (v/v) GelRed (Biotium, USA) and visualized with transilluminator (ChemiDoc^{TM} XRS+, Bio-Rad, USA). A Gene Ruler DNA ladder (Takara, China) was run as a molecular-size marker in one of the lanes. The AP-PCR fingerprints were analyzed by side-by-side visual comparison. Fingerprints were considered identical when all major bands were the same.

Supplementary Tables and Figures

Table S1. Clinical *S. mutans* and *S. sobrinus* isolates used in this study.

	Strain No.	Source	Genotyping results
S. sobrinus	1, 2, 3, 10, 11	One strain was isolated from each sample	Different genotypes
	(4, 5); (6, 7); (8, 9)	The pair in brackets are two strains isolated from the same sample	The pair in brackets are the same genotype
S. mutans	14, 17, 18 21, 28, 33, 34, 39, 44, 45	One strain was isolated from each sample	Different genotypes
	(12, 13); (15, 16); (19, 20); (29, 30); (31,32); (35, 36); (40, 41); (42, 43)	The pair in brackets are two strains isolated from the same sample	The pair in brackets are the same genotype
	(37, 38); (46, 47)	The pair in brackets are two strains isolated from the same sample	The pair in brackets are different genotypes
	22, 23, 24	Three strains isolated from the same sample	22 and 23 are the same genotype, but different from 24
	25, 26, 27	Three strains isolated from the same sample	Different genotypes

Table S2. MBC of ClyR against clinical strains isolated from SECC-children

S. mutans						*S. sobrinus*	
Strains	MBC (μg/ml)	Strains	MBC (μg/ml)	Strains	MBC (μg/ml)	Strains	MBC (μg/ml)
12	500	24	500	36	125	1	125
13	500	25	500	37	500	2	250
14	250	26	>1000	38	125	3	250
15	500	27	250	39	125	4	250
16	500	28	250	40	500	5	500
17	500	29	>1000	41	500	6	500
18	1000	30	>1000	42	500	7	500
19	250	31	125	43	250	8	250
20	500	32	500	44	500	9	250
21	250	33	500	45	250	10	250
22	500	34	250	46	250	11	500
23	250	35	125	47	250		

Figure S1 The activity of ClyR in human saliva

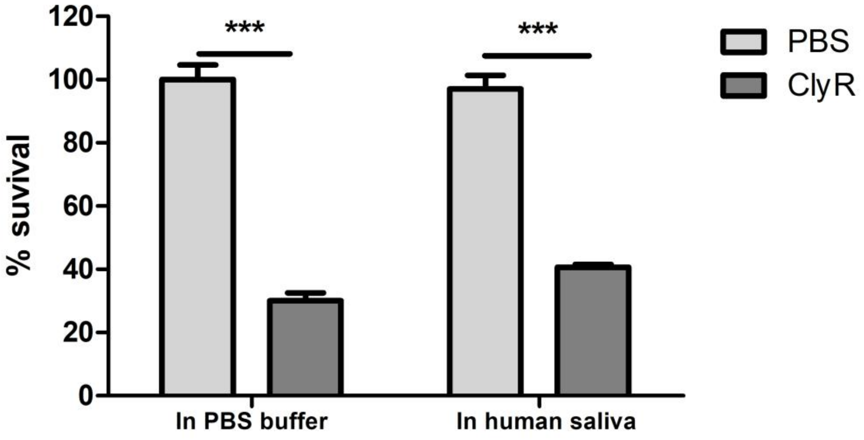

Figure S1. The activity of ClyR in human saliva. *S. mutans* MT8148 resuspended in PBS buffer or human saliva were treated with 25 µg/ml of ClyR for 1 h at 37°C, then were serially diluted and plated onto BHI agar for counting. The Y-axis represents the bacteria survival rate of each group. ***p<0.001.

Figure S2 Genotyping the clinical *S. sobrinus* (a) and *S. mutans* (b) isolates by AP-PCR.

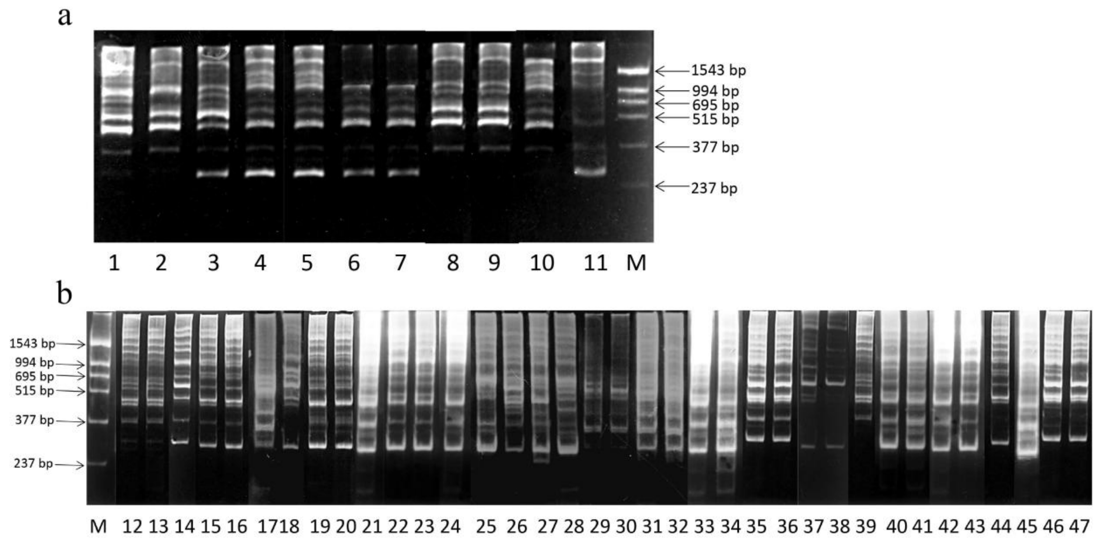

Supplement: Supplementary file 1 [file viruses-10-00380-s001.pdf]
